# Construction and validation of safe *Clostridium botulinum* Group II surrogate strain producing inactive botulinum neurotoxin type E toxoid

**DOI:** 10.1038/s41598-022-05008-1

**Published:** 2022-02-02

**Authors:** Maria B. Nowakowska, Katja Selby, Adina Przykopanski, Maren Krüger, Nadja Krez, Brigitte G. Dorner, Martin B. Dorner, Rongsheng Jin, Nigel P. Minton, Andreas Rummel, Miia Lindström

**Affiliations:** 1 Department of Food Hygiene and Environmental Health, Faculty of Veterinary Medicine, University of Helsinki, Helsinki, Finland; 2 Institut Für Toxikologie, Medizinische Hochschule Hannover, Hannover, Germany; 3 Biological Toxins, Centre for Biological Threats and Special Pathogens, Robert Koch Institute, Berlin, Germany; 4 Department of Physiology and Biophysics, University of California, Irvine, CA USA; 5 Clostridia Research Group, BBSRC/EPSRC Synthetic Biology Research Centre (SBRC), School of Life Sciences, Biodiscovery Institute, University of Nottingham, Nottingham, UK

**Keywords:** Applied microbiology, Bacteriology, Bacterial toxins, Genetic engineering, Pathogens, Food microbiology

## Abstract

Botulinum neurotoxins (BoNTs), produced by the spore-forming bacterium *Clostridium botulinum*, cause botulism, a rare but fatal illness affecting humans and animals. Despite causing a life-threatening disease, BoNT is a multipurpose therapeutic. Nevertheless, as the most potent natural toxin, BoNT is classified as a Select Agent in the US, placing *C. botulinum* research under stringent governmental regulations. The extreme toxicity of BoNT, its impact on public safety, and its diverse therapeutic applications urge to devise safe solutions to expand *C. botulinum* research. Accordingly, we exploited CRISPR/Cas9-mediated genome editing to introduce inactivating point mutations into chromosomal *bont/e* gene of *C. botulinum* Beluga E. The resulting Beluga Ei strain displays unchanged physiology and produces inactive BoNT (BoNT/Ei) recognized in serological assays, but lacking biological activity detectable *ex-* and in vivo. Neither native single-chain, nor trypsinized di-chain form of BoNT/Ei show in vivo toxicity, even if isolated from Beluga Ei sub-cultured for 25 generations. Beluga Ei strain constitutes a safe alternative for the BoNT research necessary for public health risk management, the development of food preservation strategies, understanding toxinogenesis, and for structural BoNT studies. The example of Beluga Ei generation serves as template for future development of *C. botulinum* producing different inactive BoNT serotypes.

## Introduction

Botulinum neurotoxins (BoNTs) are the most potent natural toxins known to humankind and cause botulism, a rare but potentially fatal paralytic disease affecting both humans and animals^1^. BoNTs are produced mainly by *Clostridium botulinum*, a Gram-positive, strictly anaerobic spore-forming bacterium divided into four Groups I-IV, which all harbor *bont* gene clusters^2^. Human botulism is predominantly caused by BoNTs produced by Groups I and II strains which are therefore of special concern for public health and safety^3^. Consumption of inappropriately produced or handled foods, in which *C. botulinum* had the opportunity to multiply and produce BoNT, causes food borne botulism^4^, whilst intestinal colonization with *C. botulinum* of infants and adults with bowel abnormalities or compromised intestinal microbiota results in infant/intestinal botulism. Wound botulism can result from growth and toxin production by *C. botulinum* in infected deep wounds providing an anaerobic growth milieu^5^. On the other hand, BoNT-based medicines are successfully applied in the treatment of multiple neurosecretory disorders and widely used for cosmetic purposes^6^.

The family of BoNT molecules, belonging to the zinc metalloprotease family, is divided into nine serotypes from A to G, an F/A hybrid serotype called H, and X, with a number of sub-serotype variants called subtypes^7–11^. Despite being serologically different, BoNTs share an overall sequence homology of ~ 35% and structural similarity^12^. All *C. botulinum* strains produce BoNT as a single 150-kDa polypeptide chain which is then cleaved by clostridial proteases into an active di-chain BoNT. However, the non-proteolytic Group II strains, which can produce BoNT types B, E, and F, do not produce these proteases and their BoNTs are cleaved and activated by exogenous proteases^3^. The di-chain BoNT comprises a 100-kDa heavy chain (HC) and 50-kDa light chain (LC), covalently linked by a disulfide bond^13^. Upon absorption into the body and reaching the cholinergic nerve terminals, the HC initiates the receptor-mediated endocytosis of BoNT into the nerve cells^14^. Subsequently, the catalytically active LC, an M27 Zn^2+^-dependent metalloprotease, cleaves SNARE proteins responsible for fusing acetylcholine-containing vesicles with the synaptic membrane, thus causing flaccid paralysis by blocking acetylcholine release^15^. The LCs of different BoNT serotypes and the closely related LC of tetanus neurotoxin (TeNT) have been crystallized allowing in-detail characterization of their catalytic domains^16^. Their crucial zinc-binding site contains the strictly conserved amino acid motif HExxH + E, present in all BoNT types, in TeNT, and in thermolysin, representing the well-characterized prototypical member of the gluzincin metalloprotease superfamily^17,18^. Mutational studies on BoNT/A, BoNT/B, BoNT/E and TeNT determined specific roles of the amino acids within the HExxH + E motif and its close proximity in exerting the proteolytic activity of BoNT. The two conserved histidines in the HExxH motif and the glutamic acid localized ~ 35 residues downstream of HExxH, bind Zn^2+^, while the glutamic acid in HExxH (BoNT/A E224) interacts with a water molecule essential for substrate hydrolysis^19–21^. Subsequent studies showed the importance of two other conserved amino acids, arginine (R363) and tyrosine (Y366) downstream the HExxH + E motif in stabilizing transition state intermediates (RxxY motif)^22–24^. Double mutations E224Q/Y366F or R363A/Y366F were both sufficient to reduce the catalytic activity of BoNT/A-LC below the detection threshold^20,22,25,26^. Finally, a full-length, catalytically inactive BoNT/Ai carrying the mutations E224Q within the HExxH motif and R363A and Y366F within the RxxY motif displayed no detectable neurotoxicity in the mouse phrenic nerve hemidiaphragm (MPN) assay at 30,000-fold higher concentrations than wild-type BoNT/A^27^. Crystallography confirmed that these triple mutations (E224Q/R363A/Y366F) do not alter the tertiary structure of BoNT/Ai relative to the wild-type protein and therefore circumvent a problem occurring when exchanging the Zn^2+^-binding histidine residues. Consequently, BoNT/Ai was used to resolve the crystal structure of the medium-sized (290 kDa) complex comprising BoNT/Ai and its non-toxic non-hemagglutinin NTNH^27^. Altogether, these studies provide a solid base for constructing inactive BoNT mutants of other serotypes, as their catalytic motifs are strictly conserved^18,28^.

BoNT, being the most potent natural toxin, is classified by the United States Centers for Disease Control and Prevention (CDC) as a Select Agent posing a risk to national security^29^. Therefore, *C. botulinum* research falls under strict US governmental regulations requiring specialized containment facilities and professionally trained staff to ensure maximum safety and to prevent accidents or misuse^30,31^. The extreme toxicity of *C. botulinum*-containing samples warrants restrictions for numerous research methods, especially those forming aerosols which can cause inhalational botulism^32^. Many facilities providing research services are reluctant to work with BoNT-containing samples or require its inactivation. Accordingly, there is a necessity to acquire safe surrogate strains to ensure further *C. botulinum-*focused research.

The spores of *C. botulinum* Group II pose a high risk of causing foodborne botulism due to their omnipresence in the environment and ability to survive pasteurization and germinate into toxigenic cultures at refrigeration temperatures^4^. Packaged ready-to-eat chilled food products with extended shelf lives are of particular concern^33^. Many traditional preservation strategies, such as acidification and salt^34,35^, or the use of preservatives, are rejected by modern consumers. Development of novel risk management strategies and safe products relies on challenge testing, which is laborious, expensive, and available in few food laboratories only due to strict laboratory biosafety regulations required when handling BoNT-contaminated samples. To make challenge tests more feasible, non-pathogenic *Clostridium* spp. were proposed as surrogates for *C. botulinum* in food safety testing^36^. However, there is lack of evidence of sufficient genetic or physiological relationship between the non-toxigenic *Clostridium* spp. and *C. botulinum* Group II strains, limiting the reliability of *Clostridium* spp. as surrogates. Another approach in circumventing the toxicity problem was *bont/e* gene deletion utilizing an allelic exchange approach^37^. The resulting strain displayed no toxicity in mouse bioassay (MBA) and unchanged physiology in comparison to the wild type. Nevertheless, while the construction of *bont* knockout strains may constitute an advantage for studying spore resistance and basic metabolism, it belies studies of *bont* gene expression or BoNT-complexes and structures, physiological processes interlinked with BoNT production, and validation of diagnostic methods. Additionally, toxin production and other physiological processes in *C. botulinum* appear linked^38,39^, which render *bont* knockout strains unreliable physiological surrogates.

Considering the extreme toxicity of BoNT, its potential influence on the public health and safety, as well as therapeutic applications^6^, it is important to acquire a safe strain producing an inactive form of BoNT to apply a more comprehensive approach in *C. botulinum* studies. Recently, the CRISPR/Cas9 genome manipulation method combined with homology-directed repair (HDR) was successfully implemented in *C. botulinum* Group I and Group II allowing gene deletions as well as single-nucleotide alterations^40–42^. Therefore, it constitutes a promising tool for constructing a safe *C. botulinum* strain producing inactive, but detectable BoNT.

Here we constructed and rigorously validated a surrogate *C. botulinum* Group II strain named Beluga Ei producing biologically inactive BoNT/E (BoNT/Ei). Using the CRISPR/Cas9 gene editing tool, we introduced point mutations causing amino acid substitutions within the conserved catalytic domains of BoNT/E: E213A within the HExxH motif, and R348A and Y351F within the RxxY motif. The constructed Beluga Ei strain displays unaffected growth, cell morphology, spore heat resistance, and production of BoNT/Ei compared to the Beluga wild-type strain (Beluga WT). We confirmed in vivo and ex vivo that Beluga Ei expresses inactive BoNT/Ei. Further, we showed in vivo that BoNT/Ei produced by the 25^th^ generation of the sub-cultured Beluga Ei strain is still non-toxic, validating the stability of the introduced genome modifications. Immune-based methods confirmed that BoNT/Ei displays immunogenicity comparable to the active WT BoNT/E. This work provides a safe *C. botulinum* Group II Beluga Ei strain that allows secure research on many aspects of BoNT biology and *C. botulinum* Group II physiology. This study also constitutes a solid base for future modifications of BoNT genes in other *C. botulinum* strains.

## Results and discussion

### Construction of the *C. botulinum* Beluga Ei toxoid strain using CRISPR/Cas9

The constructed *C. botulinum* Beluga Ei strain contains a chromosomally located *bont/ei* encoding enzymatically inactive toxin BoNT/Ei with three amino acid substitutions in the LC. We designed these modifications based on previous crystallography and functional studies of non-toxic recombinant variants of BoNT^19,20,22,27^. Because all BoNT serotypes demonstrate strict conservation of the catalytic motifs^18,28^, we were able to localize the previously determined residues within the BoNT/E polypeptide by sequence alignment and crystal structure analysis. Utilizing the CRISPR/Cas9 tool^41^, we introduced point mutations into the genome of the Beluga Ei strain, altering the amino acid residues of the zinc-binding motif (^638^A > C resulting in E213A within HExxH + E motif) and the transition state stabilization motif (^1042^A > G, ^1043^G > C, ^1044^G > A, ^1052^A > T resulting in R348A and Y351F within RxxY motif). In addition to these BoNT-inactivating alterations, we introduced silent modifications into the Cas9-targeted sequence of *bont/e*. All modified DNA regions were designed to introduce unique restriction sites to allow rapid discrimination between Beluga WT and Beluga Ei by PCR amplification of the modified region followed by restriction enzyme digestion. The resulting mutant clones were screened for the presence of the desired genome changes. The restriction digestion of the modification-harboring amplicon as well as Sanger and Illumina sequencing confirmed the successful modification of the *bont/ei* sequence.

Altogether, we show that CRISPR/Cas9 is a convenient tool for introducing chromosomal single-nucleotide alterations into *C. botulinum*. Our approach can be used to generate a library of *C. botulinum* strains producing other inactive BoNT serotypes. The Beluga Ei strain can be further genetically modified using CRISPR/Cas9 or any other gene manipulation tool and may serve as a base for safe research of almost every aspect of *C. botulinum* Group II physiology, including BoNT gene expression and its regulation, as well as mechanistic and structural research.

### General characterization of the *C. botulinum* Beluga Ei strain

**Figure 1:**
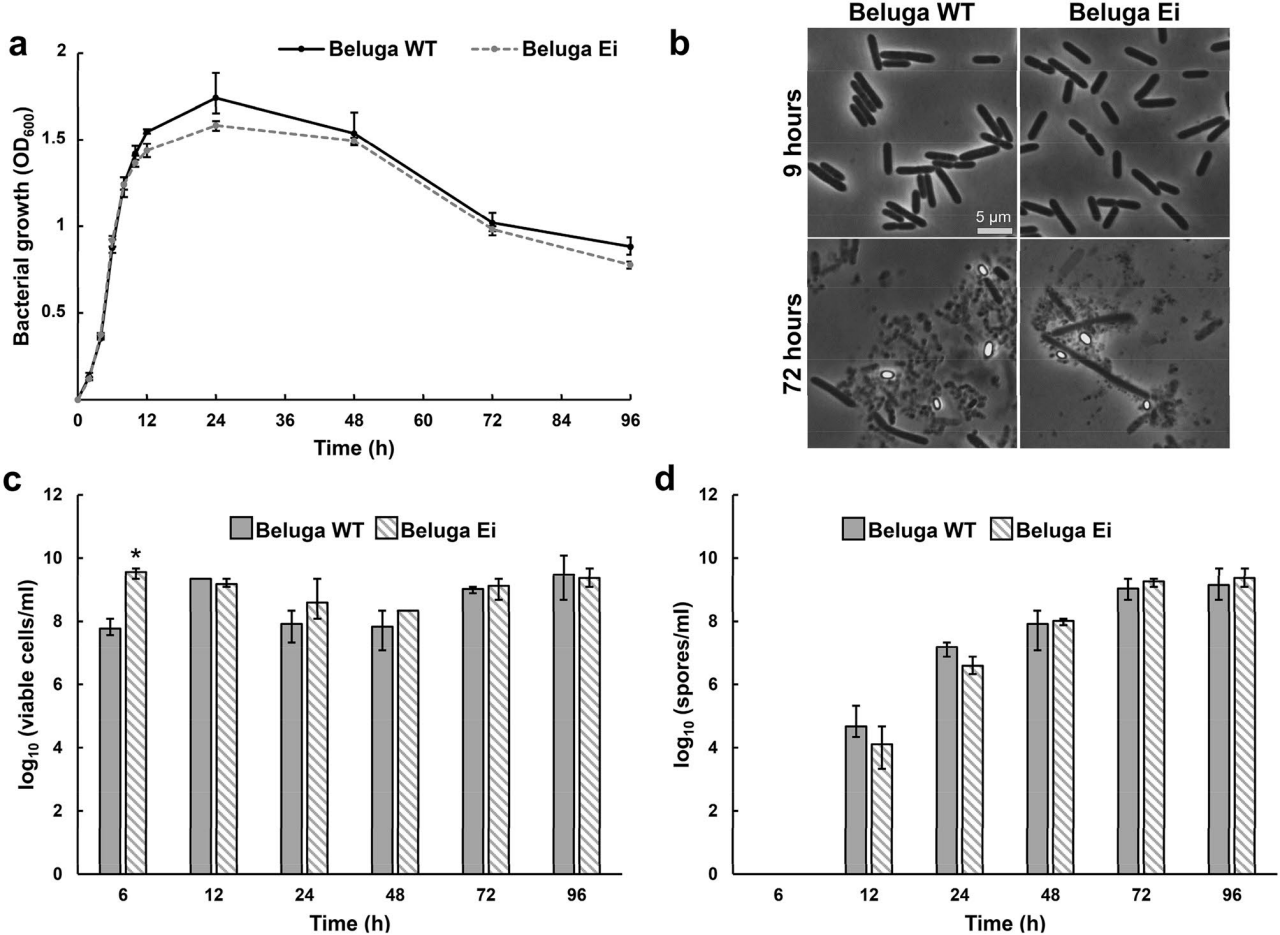
General characterization of *Clostridium botulinum* Beluga Ei strain in comparison to *C. botulinum* Beluga wild-type (Beluga WT) cultivated in cooked meat medium-tryptone-peptone-glucose-yeast extract (CMM-TPGY) broth during 96 h. (**a**) Optical density of the cultures measured at 600 nm wavelength (OD_600_). (**b**) Morphology of bacterial cells evaluated with phase-contrast microscopy during the late-logarithmic growth phase (9 h) and the late stationary phase (72 h). Concentrations of Beluga WT and Beluga Ei viable cells (**c**) and heat-resistant spores (**d**) in the culture measured during the bacterial growth. All data were obtained from three parallel biological replicates. *, cell concentration of a mutant was significantly (p < 0.05) higher than that of WT. Error bars indicate the highest and the lowest values of three biological replicates measured.

The *C. botulinum* Beluga Ei strain can serve as a reliable surrogate strain for future studies on the overall physiology and toxinogenesis of Group II strains, which are of major concern for the modern food industry. To demonstrate the applicability of the *C. botulinum* Beluga Ei strain for bacterial physiology studies, we characterized its ability to grow, display regular cell morphology, form heat-resistant spores, and produce and release detectable BoNT/Ei. These steps aimed at verifying that the introduced genomic modifications do not impact key physiological processes of the strain and, most importantly, do not affect BoNT/Ei production and its recognition in immune-based detection assays. For this purpose, we performed an in-depth characterization of growing cultures of Beluga Ei and parental Beluga WT strains in cooked meat medium-tryptone/peptone/glucose/yeast extract (CMM-TPGY) sporulation Group II medium^42^ for 96 h. The growth curves of Beluga Ei and WT showed comparable shapes and reached similar optical density (OD_600_) values, confirming that the introduced mutations in Beluga Ei did not affect its growth (Fig. 1a). Phase-contrast micrographs of late-logarithmic (9 h) and late-stationary phases (72 h post inoculation) did not reveal any morphological differences between the mutant and parental strain (Fig. 1b). Also concentrations of viable cells and heat-resistant spores were similar at most sampling time points (Figs. 1c, d). Only 6 h post inoculation, Beluga Ei demonstrated nearly 2-log higher viable cell counts than Beluga WT (Fig. 1c). However, cell division in the mid-logarithmic phase is rapid and the difference likely results from a minor delay in the sampling procedure.

**Figure 2:**
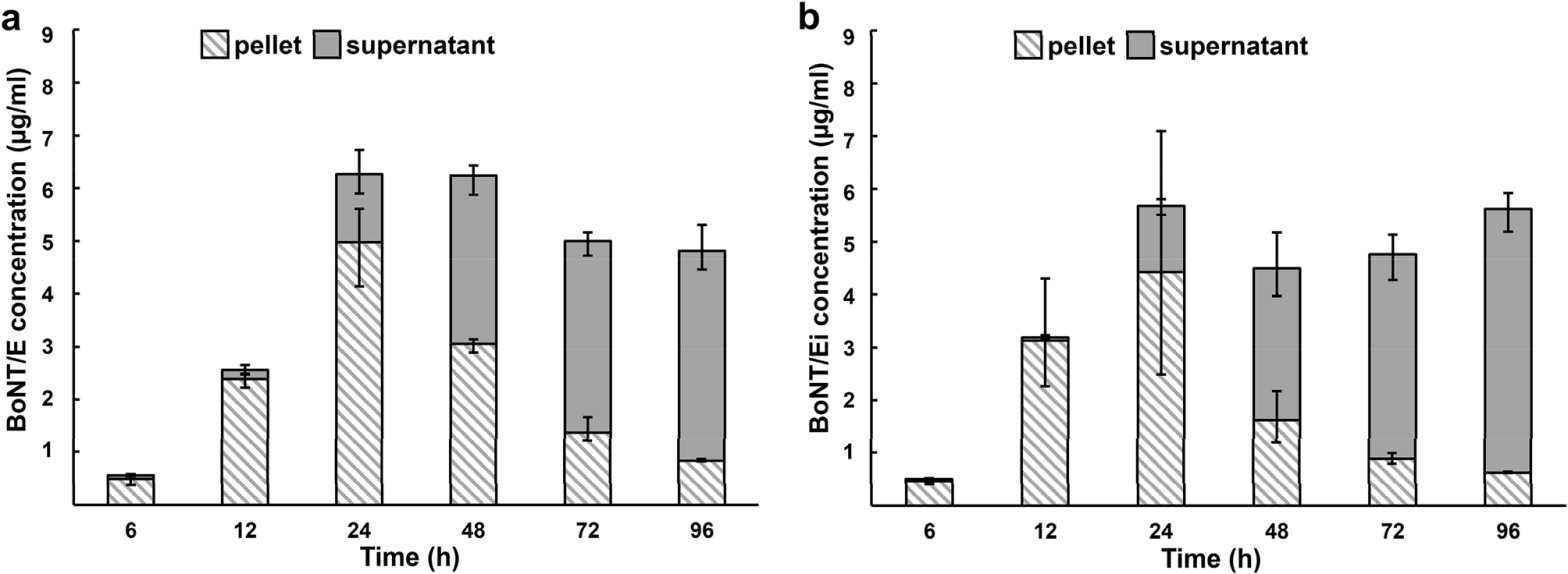
BoNT/E and BoNT/Ei concentrations and distribution in *Clostridium botulinum* Beluga wild-type (Beluga WT) (**a**) and Beluga Ei (**b**) cultures determined with BoNT/E-directed sandwich enzyme-linked immunosorbent assay (ELISA). Error bars indicate the highest and the lowest concentrations measured of the three parallel biological replicates.

Next, we verified the strains for BoNT production. We determined the concentrations of BoNT/Ei and BoNT/E in intracellular and extracellular culture fractions, respectively, using BoNT/E-directed sandwich ELISA based on the polyclonal capture antibody KE97 and the monoclonal BoNT/E-specific antibody E136 directed against the H_N_-domain of the toxin, unaltered in BoNT/Ei^43^. We additionally confirmed the ELISA-measured concentrations in densitometric analysis (Fig. S1). The antibodies applied in ELISA recognized BoNT/Ei at comparable levels to BoNT/E produced by Beluga WT (Fig. 2). The distribution of BoNT between the cell pellet and supernatant (S/N) collected at different time points was similar between the strains, confirming that the modifications within BoNT/Ei did not alter its release from the cells to the S/N. In conclusion, the introduced genome modifications did not detectably affect central cellular processes or toxin production of Beluga Ei. This successful validation provides strong evidence for the safe applicability of *C. botulinum* Beluga Ei in comprehensive studies, such as food challenge testing.

### Verification of biological activity of BoNT/Ei ex vivo and in vivo and genetic stability of *C. botulinum* Beluga Ei strain

To verify that the BoNT/Ei produced by the Beluga Ei strain is biologically inactive, we challenged the single chain BoNT/Ei (scBoNT/Ei) ex vivo using the MPN assay representing the physiological BoNT target and allowing detailed studies on BoNT pharmacodynamics^44^. Firstly, the scBoNT/E wild-type produced by Beluga WT strain was tested in the MPN assay and yielded a mean paralytic half-time of 74.5 ± 8.9 min (mean ± SD; n = 4) corresponding to 151 ± 13% neurotoxicity of recombinantly expressed, single-chain BoNTE wild-type (scBoNTE). Hence, recombinantly expressed scBoNTE wild-type and native scBoNT/E wild-type from Beluga WT display virtually identical neurotoxicity. In contrast, the samples containing scBoNT/Ei produced by the Beluga Ei strain showed no sign of paralysis of the hemidiaphragm that could be observed within the lifetime of the tissue (> 300 min). Any theoretical residual biological activity of scBoNT/Ei is well below the limit of detection of the MPN assay (LoD = 0.5 pM scBoNT/E wild-type).

The CRISPR/Cas9 approach allowed us to construct a markerless *C. botulinum* Beluga Ei strain free from external coding sequences which might display unpredictable genetic recombination events. Nevertheless, the nature of *C. botulinum* genetics has to be considered when assessing the safety of genetically modified non-toxic strains encoding specific point mutations in their genome. *C. botulinum* genomes harbor numerous mobile genetic elements and prophages that could unpredictably rearrange the BoNT cluster sequence^3,8^. Additionally, the existence of numerous different BoNT sero- and subtypes is indicating that spontaneous alterations within *bont* can occur^45^. Considering the extreme toxicity of BoNT, it is therefore crucial to verify the stability of the introduced mutations in any novel non-toxic *C. botulinum* strain to ensure its safe use.

**Figure 3:**
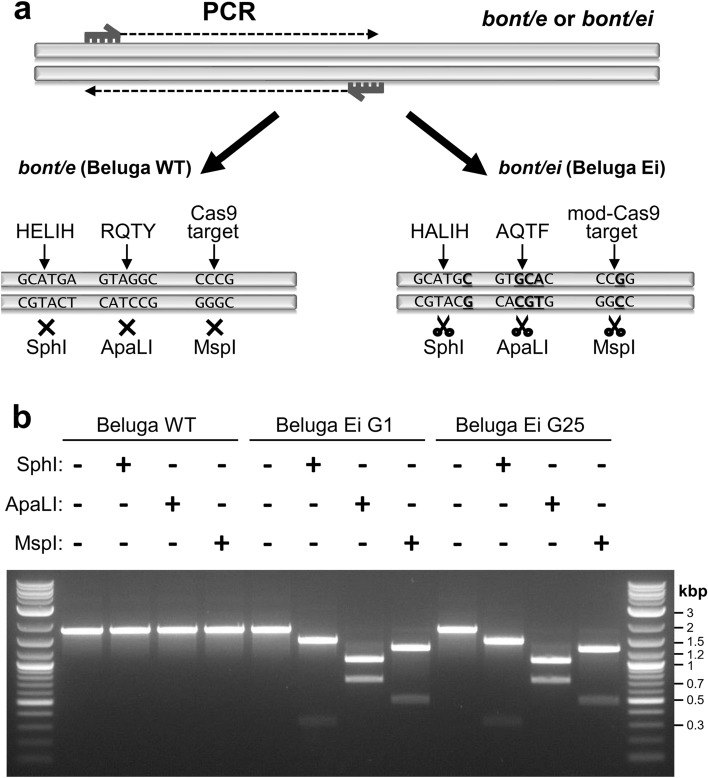
Restriction digestion-based verification of the presence of designed nucleotide modifications within *bont* locus of *Clostridium botulinum* Beluga wild-type (Beluga WT), Beluga Ei 1^st^ generation (Beluga Ei G1) and Beluga Ei 25th generation (Beluga Ei G25). (**a**) Schematic representation of the restriction digestion-based Beluga Ei strain validation. Altered nucleotides introducing the restriction sites are underlined. (**b**) Agarose gel electrophoresis visualizing the PCR amplicons after restriction digestion. Full-length agarose gel is presented in Supplementary Figure S3.

To investigate whether the introduced mutations are stable in a long-term evolutionary scenario and therefore to confirm that the constructed strain will remain safe to handle, we performed 25 single-colony serial passages of *C. botulinum* Beluga Ei strain. We subjected the resulting 25^th^ generation (G25), together with the 1^st^ generation Beluga Ei (G1), to in vivo toxicity testing and verified its genetic stability. Using a restriction endonuclease digestion approach (Fig. 3a) and Sanger sequencing, we screened a randomly picked colony of G25 for the presence of the previously introduced genomic modifications in *bont/ei*. All PCR amplicons of the targeted *bont* region of Beluga WT, Beluga Ei G1 and G25 showed the expected restriction patterns after separate digestions with SphI, ApaLI and MspI (Fig. 3b). All introduced modifications within the catalytic motifs in *bont/ei* were confirmed in Sanger (Fig. S2) and Illumina sequencing (Table S1).

**Figure 4:**
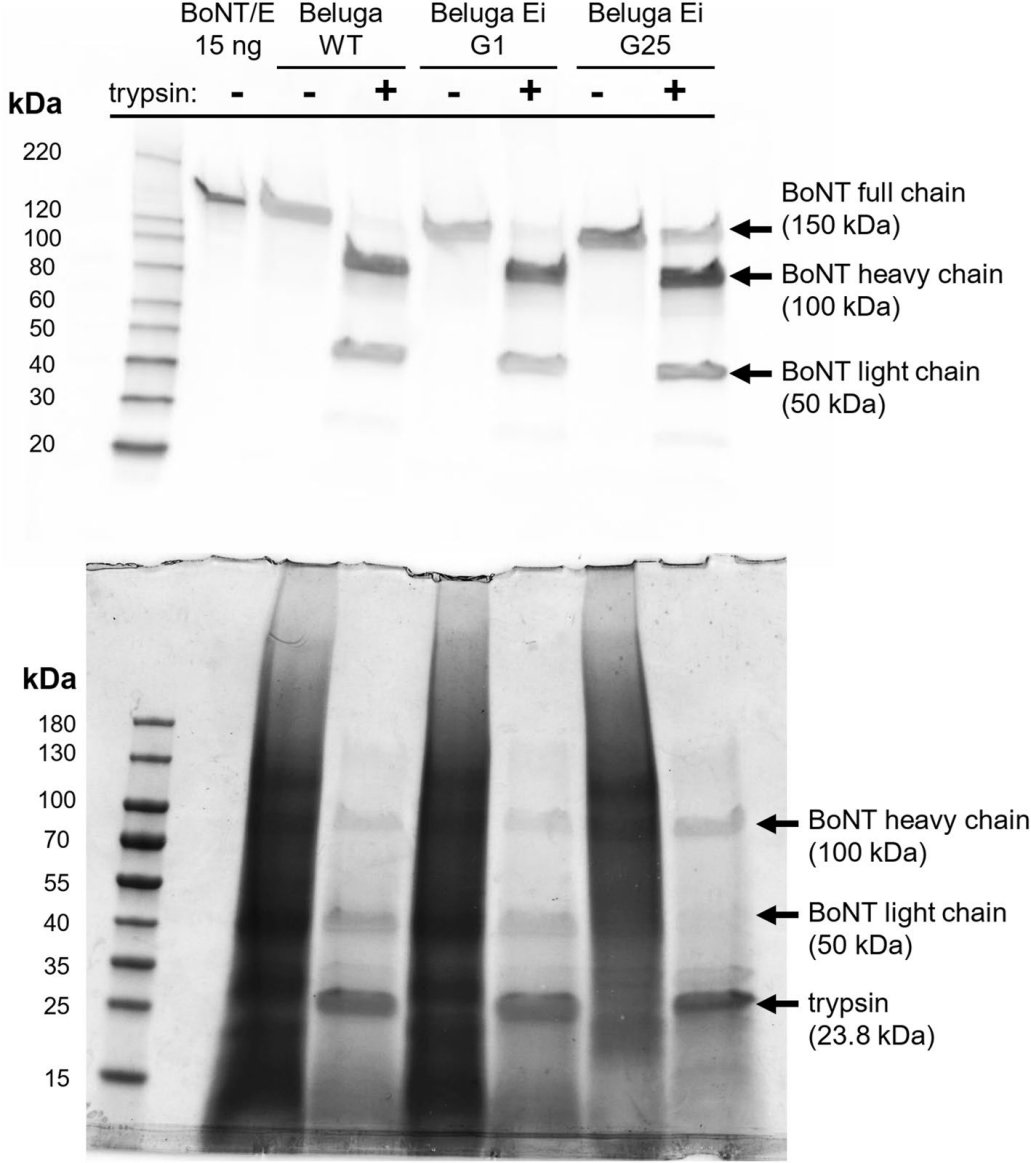
Botulinum neurotoxin (BoNT) type E-directed Western blot (upper picture) and the representative Coomassie-stained polyacrylamide gel (lower picture) validating the correct trypsin-cleavage of BoNT/E and BoNT/Ei produced by 1st and 25th generations of the *Clostridium botulinum* Beluga Ei strain (Beluga Ei G1 and Beluga Ei G25). Samples were diluted in standard phosphate-gelatin buffer prior to addition of trypsin. In the absence of trypsin gelatin creates strong background visible in Coomassie-stained gel. Full-length images of the Western blot membrane and of the Coomassie-stained gel are presented in Supplementary Figures S4 and S5, respectively.

To verify that the BoNT/Ei produced by G1 and G25 was biologically inactive, we tested the culture S/Ns in vivo using the mouse bioassay^46^. We determined the mouse intraperitoneal (i.p.) 50% lethal dose (LD_50_) of Beluga WT culture S/Ns (Table 1). After this we examined Beluga Ei G1 and G25 S/Ns to detect any residual toxicity of BoNT/Ei. We tested the BoNT toxicity of both native (scBoNT/E) and trypsinized (di-chain BoNT/E) forms, as trypsin treatment of scBoNT/E increases its i.p. toxicity more than 50-fold^47^ by cleaving the full-length 150-kDa toxin polypeptide into a 100-kDa HC and a 50-kDa LC. Western blot confirmed the efficacy of enzymatic digestion, demonstrating the expected cleavage pattern of both BoNT/E and BoNT/Ei (Fig. 4).

**Table 1:** Detection of the activity of wild-type and inactive botulinum neurotoxin type E (BoNT/E and BoNT/Ei, respectively) using the mouse bioassay.

Sample type	Sample status	Injected dose (ng/kgbw)	i.p. LD_50_ equivalent of active toxin	*n*	n*	**%
BoNT/E	Native	1333.3	5.93	2	2	100
		666.7	2.96	4	4	100
		333.3	1.48	4	4	100
		166.7	0.74	4	0	0
		133.3	0.59	2	0	0
BoNT/E	Trypsinized	13.3	5.91	2	2	100
		3.3	1.47	4	3	75
		1.7	0.76	4	1	25
		1.3	0.56	2	0	0
		0.8	0.36	4	0	0
BoNT/Ei G1	Native	107,905	480	5	0	0
	Trypsinized	53,952	23,979	5	0	0
BoNT/Ei G25	Native	104,381	464	5	0	0
	Trypsinized	52,190	23,196	5	0	0
Buffer	n.a	0	0	2	0	0

*n.a.* not applicable, *n* number of animals used; n* number of deaths, **%, percent of deaths.

Mice treated with i.p. injections of S/N preparations were monitored for any symptoms of botulism, which include “wasp-waist”, moving difficulties, respiratory problems, and death, for four days^48^. Initially, we performed an i.p. titration of trypsinized and native BoNT/E S/Ns of Beluga WT. The i.p. LD_50_ of trypsinized BoNT/E was 2.25 ng/kg of body weight (kgbw) and of native scBoNT/E was 225 ng/kgbw, as determined by the Weil’s method^49^. To assess the toxicity of BoNT/Ei G1 and G25, we administered four different S/Ns at the highest BoNT/Ei concentration that could be obtained into four groups of five mice: group i) native scBoNT/Ei G1 in amount equivalent to 480 i.p. LD_50_ of native WT scBoNT/E, ii) trypsinized BoNT/Ei G1 in amount equivalent to 23,979 i.p. LD_50_ of trypsinized WT BoNT/E, iii) native scBoNT/Ei G25 amount equivalent to 464 i.p. LD_50_ of native WT scBoNT/E, iv) and trypsinized BoNT/Ei G25 amount equivalent to 23,196 i.p. LD_50_ of WT trypsinized BoNT/E (Table 1).

During four days of observation, all the BoNT/Ei treated animals remained asymptomatic. These results confirm that the Beluga Ei strain does not produce biologically active BoNT detectable in the standard mouse bioassay and BoNT/Ei is more than 20,000-fold less toxic than WT BoNT/E thereby confirming MPN data. Moreover, BoNT/Ei produced by the 25^th^ generation of serially passaged *C. botulinum* Beluga Ei still displays no measurable biological activity. In conclusion, we demonstrated that the constructed *C. botulinum* Beluga Ei strain produces biologically inactive BoNT/Ei and that the introduced mutations within the *bont/ei* are stable for at least 25 generations, thus the likelihood of reversion to the wild-type genotype is negligible.

## Conclusions

The results demonstrate that the *C. botulinum* Beluga Ei strain, constructed in this study using the CRISPR/Cas9 tool, produces a non-toxic, biologically inactive botulinum neurotoxin structurally similar to WT BoNT/E, termed BoNT/Ei, in comparable amounts as the wild-type strain. BoNT/Ei carries three amino acid modifications in catalytically active sites: E213A in the zinc-binding HExxH + E motif^19^, and R348A and Y351F within the RxxY motif responsible for the transition state stabilization^22^, causing a loss of catalytic activity. The introduced genomic modifications did not notably affect important physiological features of the Beluga Ei strain, like growth and vegetative cell morphology, sporulation rate and the ability to form heat-resistant endospores. Most importantly, the total amount of produced BoNT/Ei and its distribution over time were similar to BoNT/E produced by the parental Beluga WT strain. Polyclonal and monoclonal antibodies directed against native BoNT/E recognized BoNT/Ei comparable to wild-type BoNT/E, and trypsin correctly cleaved the 150-kDa full-length BoNT/Ei into the expected 100-kDa HC and 50-kDa LC, indicating an unaltered BoNT/Ei tertiary structure. Mouse bioassays demonstrated absence of biological activity of trypsinized and native BoNT/Ei administered i.p. to mice in amounts equivalent to 23,979 i.p. LD_50_ of trypsinized WT BoNT/E, and to 480 i.p. LD_50_ of native WT scBoNT/E. To determine the stability of the introduced point mutations, we sub-cultured the *C. botulinum* Beluga Ei strain in 25 subsequent passages. Sanger sequencing and the restriction digestion of PCR-amplified *bont/ei* fragments of the 25^th^ mutant generation confirmed the presence of the intended mutations. Trypsinized and native culture S/N from the 25^th^ generation, containing BoNT/Ei in an amount equivalent to 23,196 i.p. LD_50_ of active trypsinized WT BoNT/E and 464 i.p. LD_50_ of native WT BoNT/E, respectively, showed no biological activity in the mouse lethality assay in groups of five mice. The results confirm that the introduced mutations within *bont/ei* are stable and reversion to the wild-type genotype is unlikely. Additionally, we confirmed the absence of detectable BoNT/Ei neurotoxicity in an ex vivo MPN assay. Here, scBoNT/Ei did not cause any sign of paralysis of the hemidiaphragm tissue at maximal achievable protein concentrations.

The constructed surrogate strain *C. botulinum* Beluga Ei carries unique restriction digestion sites as molecular markers to confirm the presence of the inactivating mutations and to facilitate identification. Being a genetically modified organism, *C. botulinum* Beluga Ei is intended exclusively for use in contained research facilities. Even in the very unlikely event of accidental release, potentially leading to Beluga Ei being erroneously detected as pathogenic *C. botulinum* strain when applying standard serological and DNA-based detection methods^50^, restriction digestion and sequencing would identify the strain as non-pathogenic. Therefore, it is important to highlight that new surrogate strains for *C. botulinum* should carry toxoid-specific molecular markers and that they must be handled only in the specialized research laboratories. In conclusion, the constructed *C. botulinum* Beluga Ei strain producing biologically inactive BoNT/Ei provides a safe and stable surrogate for botulinum neurotoxin research and for food safety risk assessment.

The *C. botulinum* Beluga Ei strain has been officially excluded from the US select agent regulations of the Division of Select Agents and Toxins (DSAT) at Centers for Disease Control and Prevention (Atlanta, GA), fulfilling all the requirements of an efficiently attenuated strain and thus not posing a threat to public health and safety (decision effective 12th December 2019 and available online^51^).

## Methods

### Strains, media, growth conditions

Strains and plasmids are listed in Table S2. The CRISPR/Cas9 plasmid pMTL431511 can be sourced at http://www.plasmidvectors.com. *C. botulinum* was cultivated at 30 °C in an anaerobic workstation (MG1000 anaerobic workstation; Don Whitley Scientific Ltd., Shipley, UK) under an atmosphere of 85% N_2_, 10% CO_2_ and 5% H_2_. Spores of *C. botulinum* were germinated in TPGY broth containing 5% (w/v) tryptone, 0.5% peptone, 2% yeast extract, 0.4% glucose, and 0.1% sodium thioglycolate. The mutant colonies were selected on TPGY plates containing 1.5% (w/v) bacteriological agar, 250 µg/ml cycloserine and 15 µg/ml thiamphenicol. For characterization, the strains were grown in CMM-TPGY consisting of 10% (w/v) cooked meat medium, 0.1% glucose, 1.5% agar and a liquid TPGY phase^42^. *E. coli* NEB5-alpha was grown at 37 °C either in Luria–Bertani (LB) broth or on agar plates, both containing 25 µg/ml chloramphenicol. *E.coli* CA434 LB broth contained additionally 30 µg/ml kanamycin.

### Identification of BoNT/E catalytic amino acid residues

The BoNT/E amino acid sequence (WP_003372387.1) of Beluga WT was aligned with the BoNT/A sequence (WP_003356619.1) in MEGA7^52^ (ClustalW, gap opening penalty 10, gap extension penalty 0.1, protein weight BLOSUM^53^). The alignment showed that E213, R348 and Y351 residues of BoNT/E are homologous to the E224, R363 and Y366 of BoNT/A, respectively. The substitutions were verified employing the crystal structures of BoNT/A (3BTA.pdb), BoNT/Ai (3V0C.pdb) and BoNT/E (3FFZ.pdb)^27,54,55^.

### Construction of the mutant strain

*C. botulinum* Beluga Ei was constructed using the CRISPR/Cas9 method^41,42^. Primers are listed in Table S3. The construction of the CRISPR/Cas9 vector pMTL431511-*bont/ei* included: (i) construction of 2,900-bp modification cassette (MC) where four DNA segments carrying the described single nucleotide mutations were PCR-synthetized using Beluga WT gDNA as template and fused by splicing by overhang extension-polymerase chain reaction (SOE-PCR); (ii) and generation of a fragment encoding the single guide RNA (sgRNA) designed using CRISPR/Cas9 tool (benchling.com/) and constructed in primer dimer-PCR reaction^41,42^. (iii) Ligation was performed as previously described^42^. *E. coli* NEB5-alpha were transformed with the ligation mixture and screened in colony-PCR. Positive plasmids were Sanger-sequenced. The conjugation donor *E. coli* CA434^56^ was transformed with pMTL431511-*bont/ei*. Conjugation was performed as previously described^42^. Antibiotic-resistant *C. botulinum* colonies were screened by colony-PCR and amplicons were Sanger-sequenced. Positive clones were cured of pMTL431511-*bont/ei* as described^42^. To rapidly confirm the presence of the mutations, amplicons from colony-PCR were digested separately with SphI, ApaLI and MspI enzymes and separated in agarose gel. The initial 1,774-bp amplicon from the genome of Beluga WT remains intact after the digestions while Beluga Ei-derived fragments are cleaved by SphI (1,449-bp and 325-bp), ApaLI (1,046-bp and 728-bp) and MspI (1,273-bp and 501-bp).

### Genomic DNA extraction and whole-genome sequencing

Genomic DNA was extracted with the Wizard Genomic DNA Purification Kit (Promega, WI, USA) and sequenced using the Next Generation Sequencing platform NovaSeq6000 (Illumina, CA, USA) at Eurofins Genomics GmbH (Ebersberg, Germany) or HiSeq2000 (Illumina) at the Institute for Molecular Medicine (University of Helsinki, Finland). *C. botulinum* Beluga WT reads were mapped to a reference genome (NZ_ACSC01000002.1), Beluga G1 reads to the Beluga WT consensus sequence, and Beluga G25 to Beluga G1 consensus sequence using CLC Genomics Workbench (version 11.0.1; Qiagen, Germany). Variants were detected using the ‘Basic Variant Detector’ tool with a minimum frequency cut-off of 35%. List of single nucleotide polymorphisms and mapping summaries are presented in Tables S1 and S4, respectively.

### Optical density measurement, spore and viable cell count assays

Spores of *C. botulinum* Beluga WT and Beluga Ei were used to inoculate CMM-TPGY in three biological replicates. Culture OD_600_ was measured at a wavelength of 600 nm using a Thermo Spectronic Genesys 10 UV/Vis Spectrophotometer. Heat-resistant spore and viable cell concentrations were determined as described^42^ applying the most probable number technique^57^. One-way ANOVA was used to compare the total viable cell and spore counts.

### Phase-contrast microscopy imaging

*C. botulinum* culture pellets were re-suspended in phosphate-buffered saline (PBS) and immobilized in 1.7% agarose coated on a microscopy slide. Micrographs were captured with a DMi8 Leica microscope, equipped with an HC PL APO 100x/1.40 OIL PH3 objective and a Hamamatsu Orca Flash V2 LT camera, and analyzed using the MetaMorph software (Molecular Devices, San Jose, CA, USA).

### BoNT/E-directed sandwich enzyme-linked immunosorbent assay

Culture aliquots were centrifuged (15 min; 6800 × *g*; + 4 °C) and cell pellets re-suspended in PBS to extract the intracellular toxin. BoNT/E-directed sandwich ELISA was carried out as described^43^ employing an affinity-purified polyclonal rabbit IgG KE97 as capture agent and the biotinylated mouse monoclonal antibody E136 directed against the H_N_-domain of BoNT/E as detector^58^. Purified BoNT/E (Metabiologics, Madison, WI, USA) was used as standard.

### Mouse phrenic nerve hemidiaphragm assay (MPN)

Beluga WT and Beluga Ei were grown in TPGY, S/Ns were harvested and dialyzed against Earl’s Balanced Salt Solution (Life Technologies, Germany). Concentration of BoNT/Ei was measured by densitometry and comparison to a BSA standard. The MPN assay was performed employing 20–30 g RjOrl:SWISS mice (Janvier SA, France) as described^44^. The time required to decrease the contraction amplitude to 50% of the starting value (paralytic half-time) upon sample administration was determined. Ex vivo tests were conducted according to §4 Abs. 3 killing of animals for scientific purposes, German animal protection law (TSchG) and MHH project no. 1679. To allow comparison of the neurotoxicity of scBoNT/E from Beluga WT supernatant with recombinantly expressed scBoNTE wild-type, a logarithmic function (y(scBoNT/E; 0.1, 0.3, 0.65, 1.0 nM) = -33,96ln(x) + 306,82; R^2^ = 0.9315) was fitted to a concentration–response-curve of recombinant BoNTE wild-type.

### Genetic stability of *C. botulinum* Beluga Ei

*C. botulinum* Beluga Ei G1 was streaked on a non-selective TPGY plate and incubated for 24 h at 30 °C. Random colonies were re-streaked and incubated as before, this was repeated for 25 subsequent days. At the 25^th^ day, a random colony was picked and designated as G25.

### In vivo determination of wild-type BoNT/E intraperitoneal LD_50_ and analysis of BoNT/Ei toxicity

CMM-TPGY media were inoculated with *C. botulinum* Beluga WT and G1 in three biological replicates and incubated for 96 h. S/Ns were collected as before (4.7) and BoNT was quantified applying ELISA (Table S5). When applicable, the samples were trypsinized^59^. To determine the toxicity of wild-type S/N, the samples were initially ten-fold serially diluted in a standard phosphate-gelatin buffer of pH = 6.2 and a volume of 0.5 ml of each sample was injected i.p. into two randomly grouped four-week-old female outbread HsdWin:NMRI mice (Envigo, Horst, The Netherlands) of 20–22 g bodyweight. The animals were monitored for symptoms of botulism for four days and euthanized by an overdose of 200 mg/kg pentobarbital (Mebunat Vet, Orion Pharma, Espoo, Finland) injected i.p. or by cervical dislocation in case of reaching the humane end-point. The highest dilution leading to death of both animals was further serially two-fold diluted and administered to four mice each, following the same protocol, to enable calculation of the i.p. LD_50_ using the Weil method^49^. To detect any residual toxicity of BoNT/Ei, each sample was injected i.p. as described above into five randomly assigned mice which were then monitored for symptoms of botulism for four days. The animal experiments were performed in accordance to the Directive 2010/63/EU of the European Parliament and of the Council of 22 September 2010 on the protection of animals used for scientific purposes, the Finnish Act on the Protection of Animals Used for Scientific or Educational Purposes (497/2013) and in compliance with ARRIVE guidelines^60^. The protocols of animal experiments were approved under the license number ESAVI/1035/04.10.07/2018 by the Project Authorisation Board of the Southern Finland Regional State Administrative Agency, Hämeenlinna, Finland.

### BoNT/E-directed Western blot

Aliquots of *C. botulinum* Beluga WT and Beluga Ei culture S/Ns before and after trypsinization were separated in 4–12% SDS-PAGE. Purified 15 ng BoNT/E standard was used as a Western blot reference. Proteins were transferred onto a PVDF 0.2 µM membrane, subsequently blocked and incubated in BoNT/E-directed KE97 rabbit polyclonal antibodies (1:1,250 in PBS with 0.05% Tween 20 (PBST) with 5% bovine serum albumin) at 4 °C overnight. The membrane was washed, incubated in goat anti-rabbit IgG:HRP antibody (Bio-Rad, Hercules, CA, USA) (1:50,000 in PBST) for 2 h at RT and washed again. Subsequently, the membrane was incubated in Clarity Western solution (Bio-Rad) and chemiluminescence was detected using Fujifilm LAS-3000 Imager (Fuji Photo Film, Tokyo, Japan) applying different exposure times.

## Supplementary Information

Supplementary Information.

## Data Availability

Whole genome sequencing data are available in the National Center for Biotechnology Information database under the BioProject PRJNA751216.
