# Supplementary material for: Construction and validation of safe Clostridium botulinum Group II surrogate strain producing inactive botulinum neurotoxin type E toxoid
Source: Sci Rep. 2022 Feb 2;12:1790. doi: 10.1038/s41598-022-05008-1 (PMC8810926; doi:10.1038/s41598-022-05008-1)
Supplement: Supplementary file 1 — Supplementary Information. [file 41598_2022_5008_MOESM1_ESM.pdf]

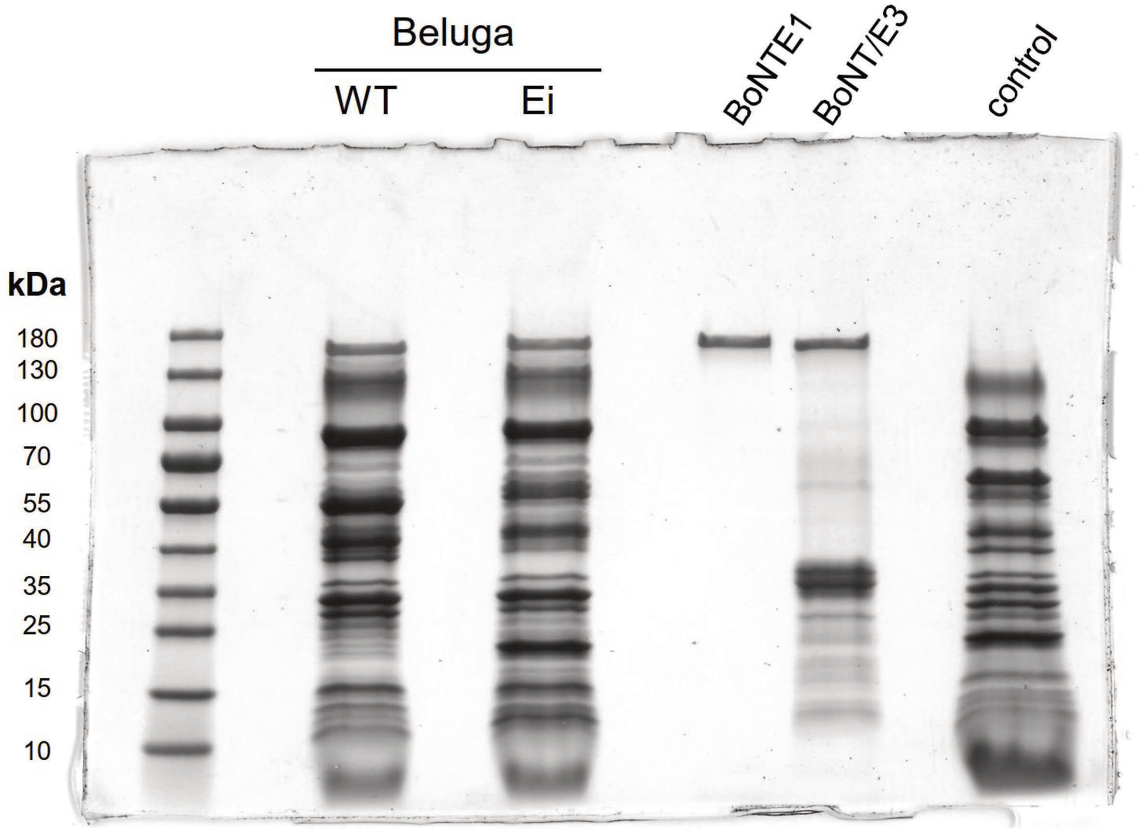


**Figure S1.** Coomassie-stained polyacrylamide gel of BoNT-containing samples. Prior to the gel electrophoresis, BoNT concentration in each sample was measured in BoNT/E-directed sandwich ELISA and volumes containing 1 µg of BoNT per lane were separated. The intensities of BoNT bands in samples from *Clostridium botulinum* Beluga WT and Beluga Ei were estimated to be similar based on densitometry image analysis (WT/Ei intensity ratio=1.08). Purified recombinant BoNTE1 and native BoNT/E3 in amounts 1 µg per lane were used as references. The control lane includes a sample from a Clostridial strain not producing BoNT.

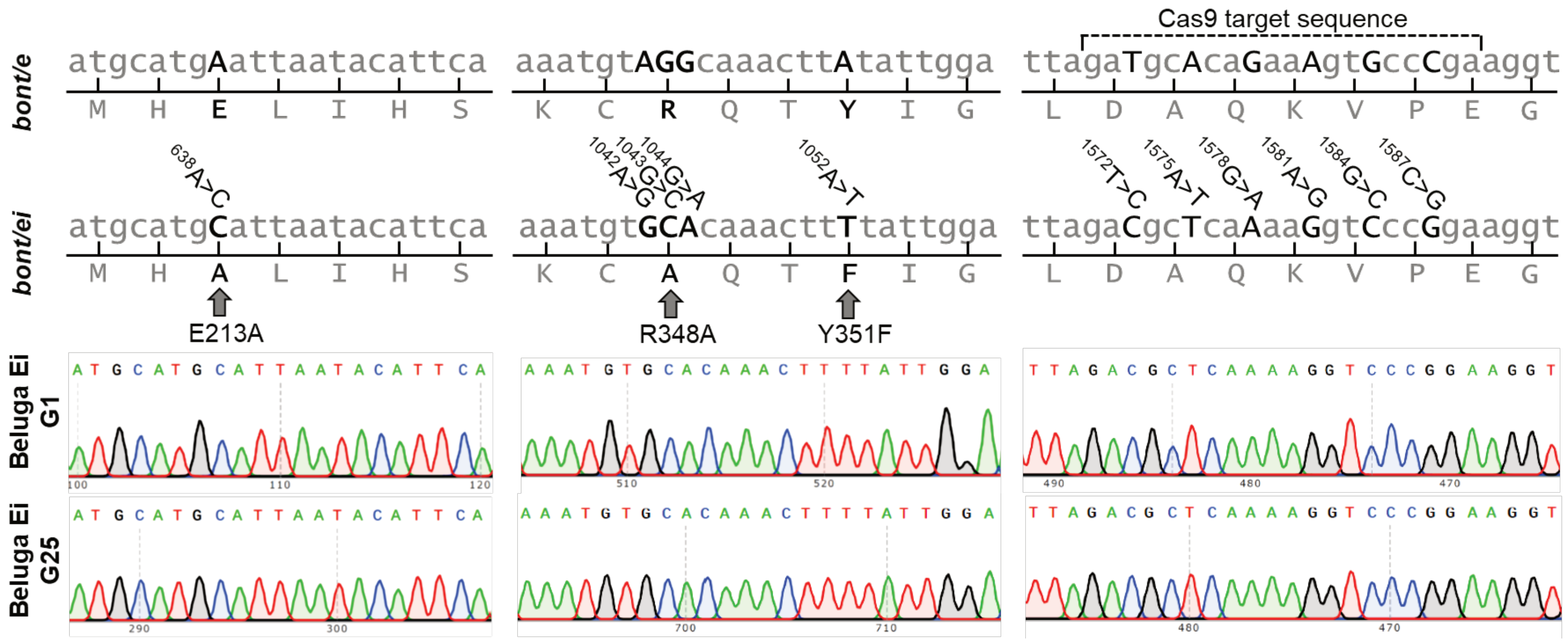


**Figure S2.** Alignment of wild-type *bont/e* and modified *bont/ei* gene sequences with Sanger sequencing chromatograms derived from 1st (Beluga Ei G1) and 25th (Beluga Ei G25) generations of *Clostridium botulinum* Beluga Ei strain. The altered base pairs and amino acids are capitalized and marked in black. The DNA sequence chromatogram of Beluga Ei G1 strain demonstrates the presence of all expected genome alterations. The DNA sequence chromatogram of Beluga Ei G25 strain still harbors all the pre-designed modifications confirming that the mutations are stably introduced into the genome for at least 25 subsequent generations.

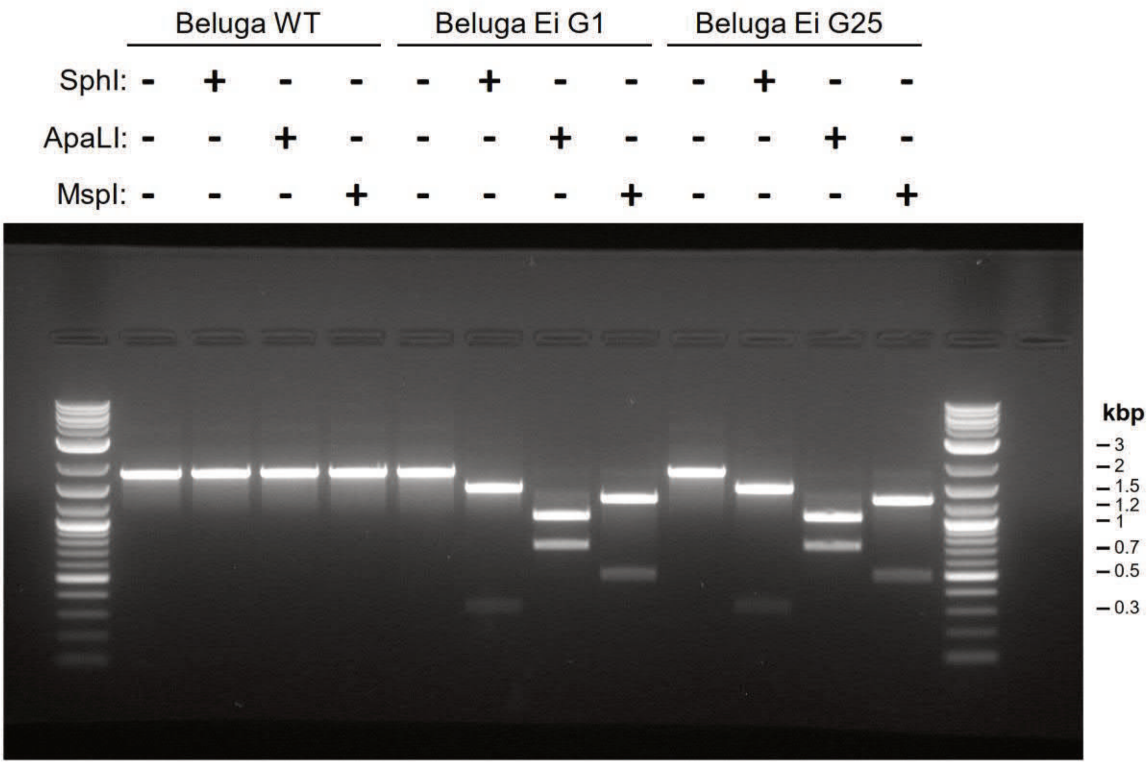


**Figure S3.** Full-length picture of the agarose gel electrophoresis visualizing the PCR amplicons after the restriction digestion. The image presents the restriction digestion-based verification of the presence of designed nucleotide modifications within *bont* locus of *Clostridium botulinum* Beluga wild-type (Beluga WT), Beluga Ei 1st generation (Beluga Ei G1) and Beluga Ei 25th generation (Beluga Ei G25).

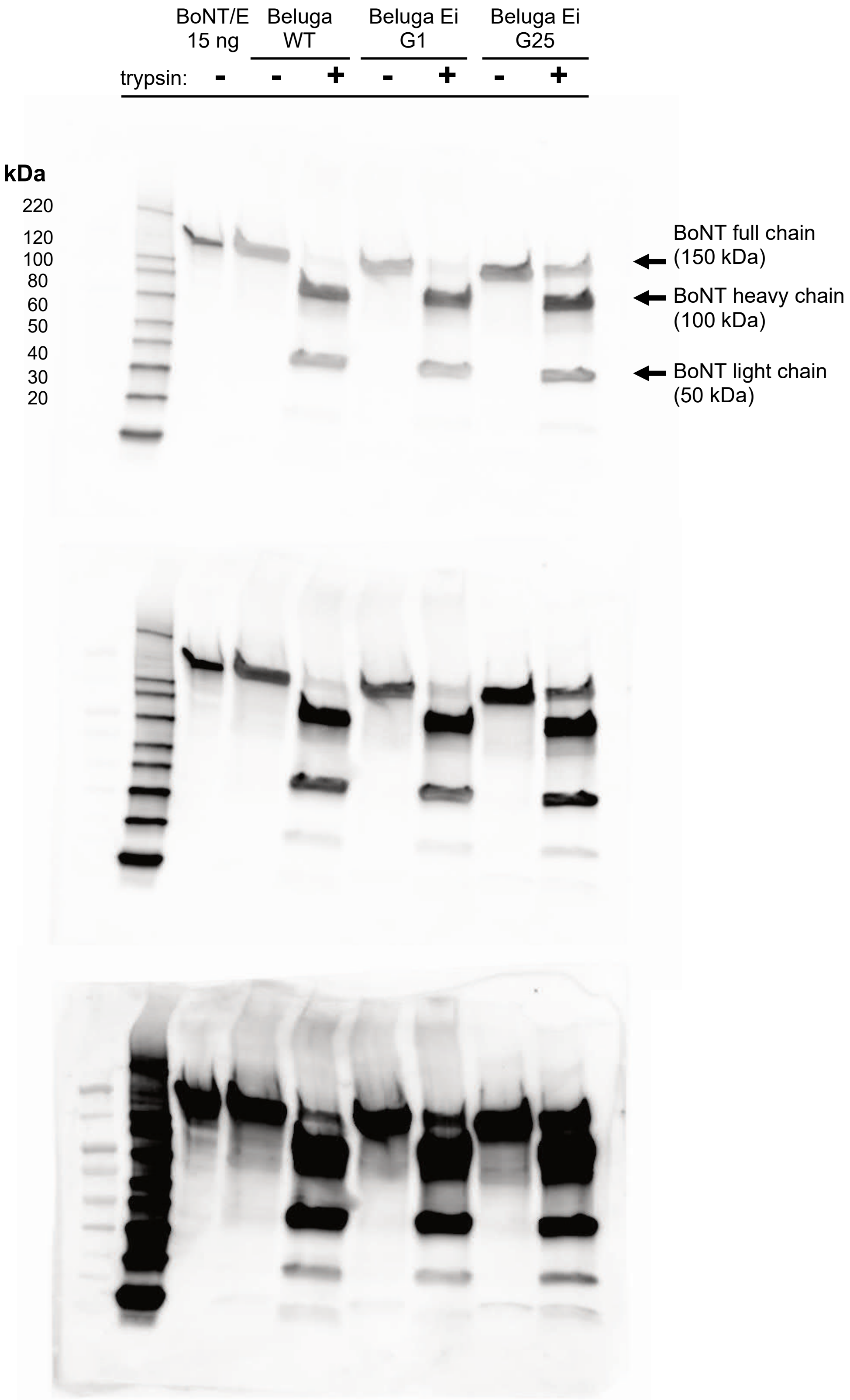


**Figure S4.** Full-length images of BoNT/E-directed Western blots presenting the validation of the correct trypsin-cleavage of BoNT/E and BoNT/Ei produced by 1st and 25th generations of the *Clostridium botulinum* Beluga Ei strain (Beluga Ei G1 and Beluga Ei G25). Upper membrane exposition time = 40 sec; middle membrane exposition time = 2 min; lower membrane exposition time = 11 min.

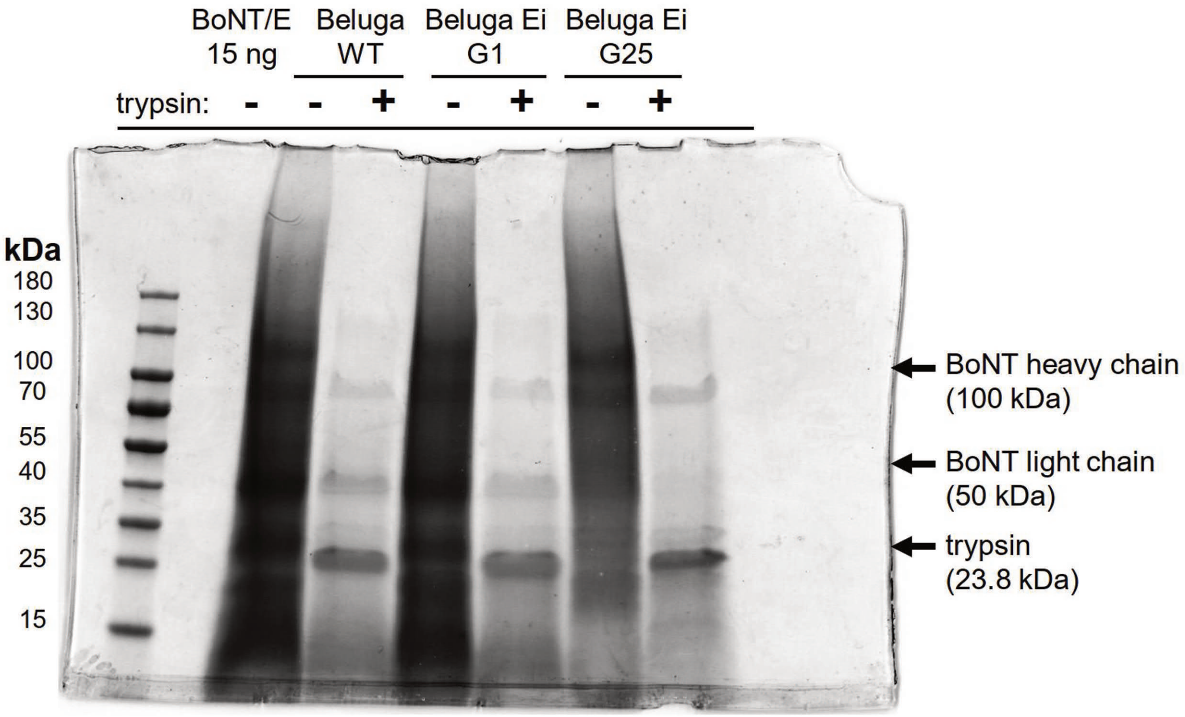


**Figure S5.** Full-length picture of Coomassie-stained polyacrylamide gel presenting the validation of the correct trypsin-cleavage of BoNT/E and BoNT/Ei produced by 1$^{st}$ and 25$^{th}$ generations of the *Clostridium botulinum* Beluga Ei strain (Beluga Ei G1 and Beluga Ei G25).

**Table S1.** List of variants detected in *C. botulinum* Beluga Ei G1 used in the present study compared to the genome sequence of Beluga WT; and the list of variants in *C. botulinum* Beluga Ei G25 compared to the genome sequence of *C. botulinum* Ei G1. Legend: AA, amino acid; CR, coding region; MNV, multiple nucleotide variation; n.a., not applicable; SNV, single nucleotide variation.

***Clostridium botulinum* Beluga Ei G1 (generation 1)**

| Coordinate | Type | Reference | Allele | Frequency (%) | CR change | AA change | Comments |
|---|---|---|---|---|---|---|---|
| 705125 | SNV | A | G | 100 | $^{1055}$A>G | Y352C | Conserved protein |
| 929871 | SNV | C | A | 99 | $^{298}$G>T | G100* | Transcriptional regulator, Fur family |
| 1097224 | SNV | G | T | 100 | n.a | n.a. | Non-coding region |
| 1217507 | SNV | G | T | 100 | $^{1582}$G>T | E528* | Type II restriction-modification system |
| 1385720 | SNV | G | T | 100 | $^{19}$G>T | A7S | LSU ribosomal protein L20p |
| 1568113 | SNV | C | A | 100 | $^{549}$G>T | n.a. | Hypothetical protein |
| 1687587 | SNV | G | T | 100 | $^{136}$G>T | A46S | Hypothetical protein |
| 2000599 | SNV | C | A | 99 | $^{847}$C>A | P283T | tRNA dimethylallyltransferase |
| 2536554 | SNV | G | T | 100 | n.a | n.a. | Non-coding region |
| 2628988 | SNV | C | A | 100 | $^{373}$G>T | A125S | Oligoribonuclease A |
| 2763800 | SNV | C | A | 100 | $^{210}$G>T | K70N | GTP-binding protein TypA/BipA |
| 2789844 | SNV | A | C | 99 | $^{638}$A>C | E213A | Neurotoxin type E (toxoid modification) |

| Coordinate | Type | Reference | Allele | Frequency (%) | CR change | AA change | Comments |
|---|---|---|---|---|---|---|---|
| 2790248 | MNV | AGG | GCA | 99 | $^{1042-1044}$AGG del<br>$^{1042-1044}$GCA ins | R348A | Neurotoxin type E (toxoid modification) |
| 2790258 | SNV | A | T | 100 | $^{1052}$A>T | Y351F | Neurotoxin type E (toxoid modification) |
| 2790778 | SNV | T | C | 100 | $^{1572}$T>C | n.a. | Neurotoxin type E (toxoid modification) |
| 2790781 | SNV | A | T | 100 | $^{1575}$A>T | n.a. | Neurotoxin type E (toxoid modification) |
| 2790784 | SNV | G | A | 89 | $^{1578}$G>A | n.a. | Neurotoxin type E (toxoid modification) |
| 2790787 | SNV | A | G | 89 | $^{1581}$A>G | n.a. | Neurotoxin type E (toxoid modification) |
| 2790790 | SNV | G | C | 96 | $^{1584}$G>C | n.a. | Neurotoxin type E (toxoid modification) |
| 2790793 | SNV | C | G | 95 | $^{1587}$C>G | n.a. | Neurotoxin type E (toxoid modification) |
| 2912731 | SNV | C | A | 100 | $^{3}$G>T | n.a. | Hypothetical protein |
| 3015605 | SNV | C | A | 100 | $^{298}$G>T | G100* | Nucleoside diphosphate kinase |
| 3423844 | SNV | C | A | 100 | $^{884}$G>T<br>$^{1118}$G>T | R295I R373I | Aminoacyl-histidine dipeptidase |
| 3461123 | SNV | C | A | 100 | $^{2042}$G>T | S681I | RecD-like DNA helicase YrrC |
| 3759932 | SNV | G | T | 100 | $^{57}$C>A | n.a | DNA integrity scanning protein DisA |

**_Clostridium botulinum_ Beluga Ei G25 (generation 25)**

| Coordinate | Type | Reference | Allele | Frequency (%) | CR change | AA change | Comments |
|---|---|---|---|---|---|---|---|
| 222801 | SNV | C | A | 99 | $^{785}$C>A | S262Y | Glucose-1-phosphate adenylyltransferase |
| 442373 | SNV | C | A | 99 | $^{560}$C>A | A187D | Choline binding protein A |
| 964192 | SNV | G | T | 99 | $^{2077}$G>T | G693C | 5-methyltetrahydrofolate-homocysteine methyltransferase |
| 1207656 | SNV | C | A | 99 | $^{1208}$C>A | A403E | Endonuclease |
| 2396875 | SNV | G | T | 99 | $^{729}$C>A | F243L | PTS system, beta-glucoside-specific IIB component |
| 2482821 | SNV | C | A | 99 | $^{1000}$G>T | E334* | Two-component response regulator |
| 3227216 | SNV | G | T | 99 | $^{22}$C>A | P8T | Hypothetical protein |
| 3279633 | SNV | C | A | 100 | $^{36}$G>T | K12N | Endonuclease IV |
| 3322036 | SNV | G | T | 99 | $^{211}$G>T | D71Y | Transcriptional regulator, DeoR family |
| 3963865 | SNV | G | T | 99 | $^{3245}$C>A | A1082E | ATP-dependent nuclease, subunit A |

**Table S2.** List of strains and plasmids used.

| Strains/plasmids | Description | Source |
|---|---|---|
| ***Clostridium botulinum*** | | |
| Beluga WT | Wild-type strain isolated from fermented Beluga whale flippers | IFR[a] |
| Beluga Ei G1 | 1st generation of the mutant containing three amino acid substitutions E213A/R348A/Y351F in *bont/e* gene | This study |
| Beluga Ei G25 | 25th generation of the mutant containing three amino acid substitutions E213A/R348A/Y351F in *bont/e* gene | This study |
| ***Escherichia coli*** | | |
| CA434 | Conjugation donor | Purdy et al. |
| CA434 + pMTL431511-*bont/ei* | Conjugation donor | This study |
| NEB 5-alpha | Cloning strain | New England BioLabs |
| **Plasmids** | | |
| pMTL431511 | Empty CRISPR/Cas9 vector | SBRC[b]. |
| pMTL431511-*bont/ei* | CRISPR/Cas9 vector for constructing Beluga Ei mutant | This study |

[a]Culture Collection of the Institute of Food Research, Norwich, UK

[b] Synthetic Biology Research Centre, Nottingham UK. CRISPR/Cas9 vectors may be sourced at http://www.plasmidvectors.com

**Table S3**. List of primers. The underlined sequences indicate the restriction enzyme cleavage sequence.

| Primer name | Sequence (5´→3´) | Information |
|---|---|---|
| F_MC1_*bont/ei*-AsiSI | TATATAGCGATCGCGGGATGAGGTAA TATTTTCTGTATTAGATG | Construction of the first segment of the modification cassette for pMTL431511-*bont/ei.* Primer used for SOE-PCR amplification of final MC. |
| R_MC1_*bont/ei* | CATGTAATGAATGTATTAATGCATGCA TTAATGTAAG | Construction of the first segment of the modification cassette for pMTL431511-*bont/ei*. Primer introduces E213A substitution into *bont/e* sequence ($^{638}$A>C). |
| F_MC2_ *bont/ei* | CTTACATTAATGCATGCATTAATACATT CATTACATG | Construction of the second segment of the modification cassette for pMTL431511-*bont/ei*. Primer introduces E213A substitution into *bont/e* sequence ($^{638}$A>C). |
| R_MC2_ *bont/ei* | CAATAAAAGTTTGTGCACATTTAACTT GAAATTTAGTTGCTAAATCAAATTCC | Construction of the second segment of the modification cassette for pMTL431511-*bont/ei*. Primer introduces R348A and Y351F substitutions into *bont/e* sequence ($^{1042}$A>G, $^{1043}$G>C, $^{1044}$G>A, $^{1052}$A>T). |
| F_MC3_ *bont/ei* | CAAGTTAAATGTGCACAAACTTTTATT GGACAGTATAAATACTTCAAACTTTC | Construction of the third segment of the modification cassette for pMTL431511-*bont/ei*. Primer introduces R348A and Y351F substitutions into *bont/e* sequence ($^{1042}$A>G, $^{1043}$G>C, $^{1044}$G>A, $^{1052}$A>T). |
| R_MC3_*bont/ei* | CGGGACCTTTTGAGCGTCTAAATAGA AAAATACATTAAGTTCATTAACATC | Construction of the third segment of the modification cassette for pMTL431511-*bont/ei*. Primer introduces six silent mutations into the CRISPR/Cas9 targeting *bont/e* sequence ($^{1572}$T>C, $^{1575}$A>T, $^{1578}$G>A, $^{1581}$A>G, $^{1584}$G>C, $^{1587}$C>G). |

| Primer name | Sequence (5´→3´) | Information |
|---|---|---|
| F_MC4_ *bont/ei* | GACGCTCAAAAGGTCCCGGAAGGTGAAAATAATGTCAATCTCAC | Construction of the fourth segment of the modification cassette for pMTL431511-*bont/ei*. Primer introduces six silent mutations into the CRISPR/Cas9 targeting *bont/e* sequence ($^{1572}$T>C, $^{1575}$A>T, $^{1578}$G>A, $^{1581}$A>G, $^{1584}$G>C, $^{1587}$C>G). |
| R_MC4_ *bont/ei*-AscI | TATATAGGCGCGCCCATATCCTGAAGTATCTACGTATTTATC | Construction of the fourth segment of the modification cassette for pMTL431511-*bont/ei.* Primer used for SOE-PCR amplification of final MC. |
| F_*bont/e*_sgRNA-SalI | TTTTCGTCGACGATGCACAGAAAGTGCCCGAGTTTTAGAGCTAGAAATAGCAAGTTAAAATAAGGCTAGTCCGTTATCAACTTGAAAAAGTGGCACCGAGTCGGTGC | Construction of a fragment containing *bont/e*-specific sgRNA template. |
| R_sgRNA-AsiSI | CGCGCGCGGCGATCGCATAAAAATAAGAAGCCTGCAAATGCAGGCTTCTTATTTTTATAAAAAAAGCACCGACTCGGTGCCACTTTTTCAAGTTG | Construction of a fragment containing sgRNA template. |
| F_ *bont/ei*_RE | GAAGAACTGTCAAAAGCTAATCCA | Amplification of modified region within *bont/e* and *bont/ei* for further restriction enzyme digestion, sequencing of pMTL431511-*bont/ei* |
| R_*bont/ei*_RE | GTGTATTAATTTTAGTCATCCAATTCG | Amplification of modified region within *bont/e* and *bont/ei* for further restriction enzyme digestion, sequencing of pMTL431511-*bont/ei* |
| F_sgRNA_seq | CTAGATTTATATTTAGTCCCTTGCC | Sequencing of pMTL431511 plasmids series. |

| **Primer name** | **Sequence (5´→3´)** | **Information** |
|---|---|---|
| 83XXX-LR | ACGGCTTGATGTGTTGGTAG | Sequencing of pMTL431511 plasmids series. |
| F_ *bont/ei*_scr | CAACTAGTAGATAATAAAAATAATGCA CAG | Screening and sequencing of Beluga Ei mutant. |
| R_*bont/ei*_scr | GATTTTTATTAGTTGGATATTTATATAC ATCTCC | Screening and sequencing of Beluga Ei mutant. |

**Table S4.** Read mapping summary report and accession numbers of sequenced *Clostridium botulinum* samples. Data were deposited under the project number PRJNA751216 in Sequence Read Archive (SRA). Beluga WT reads were mapped against the reference genome available in NCBI database. Beluga Ei G1 reads were mapped against assembled consensus sequence of Beluga WT, and Beluga Ei G25 reads mapped against Beluga Ei G1 assembled consensus sequence.

| Feature | Beluga WT | Beluga Ei G1 | Beluga Ei G25 |
|---|---|---|---|
| BioSample Accession Number | SAMN20513657 | SAMN20511721 | SAMN20511722 |
| SRA Accession number | SRR15317859 | SRR15312153 | SRR15312152 |
| **Mapping Summary** | | | |
| Total reads | 11,150,642 | 27,176,720 | 23,838,926 |
| Mapped reads | 10,820,072 | 27,124,936 | 23,727,788 |
| Unmapped reads | 330,570 | 51,784 | 111,138 |
| % mapped reads | 97.04% | 99.81% | 99.53% |
| % unmapped reads | 2.96% | 0.19% | 0.47% |

**Table S5.** Toxin concentration of the supernatant samples used in mouse bioassays measured in BoNT/E-directed sandwich ELISA. Each sample was diluted in at least 3 technical replicates.

| ***C. botulinum* strain** | **BoNT concentration (ng/ml)** | **Negative error (ng/ml)** | **Positive error (ng/ml)** |
|---|---|---|---|
| Beluga WT | 5606 | 200 | 307 |
| Beluga Ei G1 | 4532 | 553 | 600 |
| Beluga Ei G25 | 4384 | 547 | 735 |
